# Prevalence and global trends of polypharmacy in patients with chronic kidney disease: A systematic review and meta-analysis

**DOI:** 10.3389/fphar.2023.1122898

**Published:** 2023-02-10

**Authors:** Lina Naseralallah, Malkan Khatib, Azhar Al-Khulaifi, Mohammed Danjuma

**Affiliations:** ^1^ Clinical Pharmacy Department, Hamad Medical Corporation, Doha, Qatar; ^2^ School of Pharmacy, College of Medical and Dental Sciences, University of Birmingham, Birmingham, United Kingdom; ^3^ College of Medicine, QU Health, Qatar University, Doha, Qatar; ^4^ Internal Medicine Department, Hamad Medical Corporation, Doha, Qatar; ^5^ Weill Cornell College of Medicine, Doha, Qatar

**Keywords:** polypharmacy, chronic kidney disease (CKD), epidemiology, systematic review, meta-analysis

## Abstract

**Background and objectives:** Polypharmacy and chronic kidney disease (CKD) are becoming increasingly common due to an ageing population and the rise of multimorbidity. In line with the therapeutic guidelines, managing CKD and its complications necessitates prescribing multiple medications, which predisposes patients to polypharmacy. The aim of this systematic review and meta-analysis is to describe the prevalence of polypharmacy in patients with CKD and to explore the global trends of factors driving any apparent variability in prevalence estimates.

**Methods:** PubMed, Scopus, the Cochrane Database of Systematic Reviews (CDSR), and Google Scholar were searched from 1999 to November 2021. Study selection, data extraction, and critical appraisal were conducted by two independent reviewers. The pooled prevalence of polypharmacy was estimated utilizing the random effects model using the default double arcsine transformation.

**Results:** This review involved 14 studies comprising of 17 201 participants, a significant proportion of which were males (56.12%). The mean age of the review population was 61.96 (SD ± 11.51) years. The overall pooled prevalence of polypharmacy amongst patients with CKD was 69% (95% CI: 49%–86%) (I^2^ = 100%, *p* < 0.0001), with a proportionately higher prevalence in North America and Europe as compared to Asia.

**Conclusion:** The results from this meta-analysis showed a high pooled prevalence estimates of polypharmacy amongst patient cohorts with CKD. The exact interventions that are likely to significantly mitigate its effect remain uncertain and will need exploration by future prospective and systematic studies.

**Systematic Review Registration**: [https://www.crd.york.ac.uk/prospero/], identifier [CRD42022306572].

## Introduction

Chronic kidney disease (CKD) is a relatively common condition that affects up to 16% of the population globally (Kidney Disease: Improving Global Outcomes (KDIGO) CKD Work Group, 2013; Gansevoort et al., 2011). It has been associated with adverse health outcomes including cardiovascular disease (CVD) events, poor quality of life, significant morbidity, and CVD or all-cause mortality (Kidney Disease: Improving Global Outcomes (KDIGO) CKD Work Group, 2013; Couser et al., 2011; James et al., 2010; Yuan et al., 2017; Bansal et al., 2017; Jankowski et al., 2021). CKD is a gradually progressive disease that is linked to a range of complications, such as anemia, bone and mineral disorder, electrolyte imbalance, acid-base abnormalities, hypertension and other CVD, and sexual dysfunction (Widmer et al., 1979; Delmez and Slatopolsky, 1992; Stefanski et al., 1996; Hsu et al., 2002; Bello et al., 2017). Therefore, therapeutic guidelines have been developed to prevent/slow the progression of CKD and to provide therapeutic approaches to manage each clinical manifestation (Kidney Disease: Improving Global Outcomes (KDIGO) CKD Work Group, 2013). As a consequence of the advanced therapeutics that followed our rising understanding of the pathophysiology, the number of medications taken per patient has substantially increased, which made polypharmacy and its expensive consequences a commonplace across this cohort of patients.

Polypharmacy exhibits a pronounced risk for medication non-adherence, adverse drug events, problematic interactions (drug-drug, drug-food, and pharmacogenetic), emergency department visits, hospitalizations, and sometimes avoidable mortality (Whittaker and Fink, 2017). Consequent upon this, a hefty medical and financial burdens have been imposed on patients, societies, and healthcare systems (Kidney Disease: Improving Global Outcomes (KDIGO) CKD Work Group, 2013; Jankowski et al., 2021). It is not clear if this polypharmacy is appropriate, as despite the medication burden in CKD patients that could reach more than 30 drugs per patient, the morbidity and mortality rates remain high which raises questions about the effectiveness of these medications in this cohort of patients (Whittaker and Fink, 2017; Schmidt et al., 2019). Additionally, the altered pharmacodynamic and pharmacokinetic parameters in the unique milieu of renal insufficiency further complicates the situation as dosage adjustments and cessation of certain therapies might be required (Parker and Wong, 2019).

Whilst the growing polypharmacy epidemic has garnered attention in the medical community, it is still challenging to address this issue as uncertainty still exist regarding the exact prevalence of polypharmacy in CKD patients as well as the socio-demographic factors that influence its global trends. Understanding the burden of polypharmacy in patients with CKD and identifying vulnerable populations will enable clinicians to develop and implement interventions (e.g., deprescribing) to mitigate polypharmacy and its unfavorable outcomes. Therefore, the aim of this systematic review and meta-analysis is to estimate the prevalence of polypharmacy in CKD patients and to explore the global trends of factors driving any apparent variability in prevalence estimates.

## Materials and methods

### Registration and methodology reporting

This systematic review and meta-analysis followed the recommendations provided by the PRISMA (Preferred Reporting Items for Systematic reviews and Meta-Analyses) guidelines. The current review has been registered with PROSPERO under the registration number: CRD42022306572. This authorization encompasses patient cohorts with both CKD and chronic liver disease (CLD).

### Data sources and search strategy

Searches were undertaken using the following electronic databases and search engines from 1999 to November 2021: PubMed, Scopus, the Cochrane Database of Systematic Reviews (CDSR), and Google Scholar (first 50 pages). The process also included cross-referencing of included papers. The choice of these databases as primary areas of our literature search was based on their validation as repositories of the most current medical literature; in addition to their continued update and renewal.

The following search terms were used: “polypharmacy” [TIAB], AND “kidney” [MeSH] OR “CKD” OR “chronic kidney disease” [MeSH]. We additionally searched grey literature for similar articles that are not captured in the aforementioned databases. The search was limited to “English language” and “Human species” as applicable to each database.

### Eligibility criteria

This systematic review and meta-analysis examined studies of patient populations with CKD who had medication counts adjudicated as polypharmacy and reported as such. The main outcome is the pooled estimate of polypharmacy period prevalence amongst these studies. Our review does not involve a new investigational medicinal product (IMP), hence no conceivable need for an intervention and comparator arms.

Studies were considered eligible if they met the following criteria: (1) reported numerical data on the prevalence of polypharmacy; (2) included patients with CKD; (3) included participants of ≥18 years; (4) published in English language. We included studies regardless of what the authors considered to be the threshold for diagnosis of polypharmacy (e.g., ≥5, ≥10 medications, etc.). Case report, case series, and reviews were excluded as were studies that only included patients with polypharmacy (patients who have CKD without polypharmacy were excluded) as this means that the control/denominator is missing which will prevent calculating the prevalence.

### Study selection

All retrieved studies were exported to EndNote 20^®^ (2021 Clarivate), duplicates were removed, and the remaining papers were imported to Rayyan Qatar Computing Research Institute (QCRI) software. Two independent reviewers (LN and MD) screened the titles, abstracts, and full texts of the records to ascertain eligibility for inclusion. Disagreements between reviews were resolved through consensus discussion with a third reviewer (MK).

### Data extraction

Two independent reviewers (LN and MD) initially trialed a sample data collection sheet on five randomly selected studies to determine the robustness of this sheet in abstracting patient data. After which the following variables were extracted from the studies: author, year of publication, study design, site, country, population, age, gender distribution, sample size, proportion of CKD patients with polypharmacy, iteration of polypharmacy definition (where available), and duration of study.

### Quality assessment

The risk of bias of the included studies was carried out using the Loney’s criteria. Exhaustive description of this appraisal tool has been done elsewhere (Loney et al., 1998). Briefly, the tool is comprised of eight domains returning a total score of eight for studies with optimal methodological quality. Two reviewers (LN and MD) independently assessed the methodological quality. Disagreements were resolved by consensus or by involving a third reviewer (AA).

### Data synthesis

Continuous Variables were presented as means (± standard deviation [SD]) or median (interquartile range [IQR]) as appropriate; whilst categorical variables were presented as numbers (percentages). We quantified the pooled prevalence estimates of polypharmacy (utilizing the random effects model) amongst patients with CKD using the default double arcsine transformation. We did not apply any continuity correction because this transformation does not need one. To ascertain the source of significant heterogeneity amongst the included studies (where this exists), we subsequently carried out a subgroup analyses to examine the effect of age, gender, source of primary data, as well as the risk of bias scores of the reviewed studies on the final point prevalence estimates. We assessed the heterogeneity between studies with I^2^ statistic and τ^2^ statistic (Higgins et al., 2003). We considered the I^2^ thresholds of 25%, 50%, and 75% to represent low, moderate, and high heterogeneity between-study variances, respectively. Where τ^2^ was reported to be zero, this was indicative of no heterogeneity (Quintana, 2015). We utilized funnel and Doi plots to visualize small-study effect and publication bias (Furuya-Kanamori et al., 2018). We finally carried out sensitivity analysis excluding each study to ascertain its effect on the final point prevalence estimate. All statistical analyses were carried out with Meta XL, version 5.3 (EpiGear International, Queensland, Australia).

## Results

### Study selection

**FIGURE 1:**
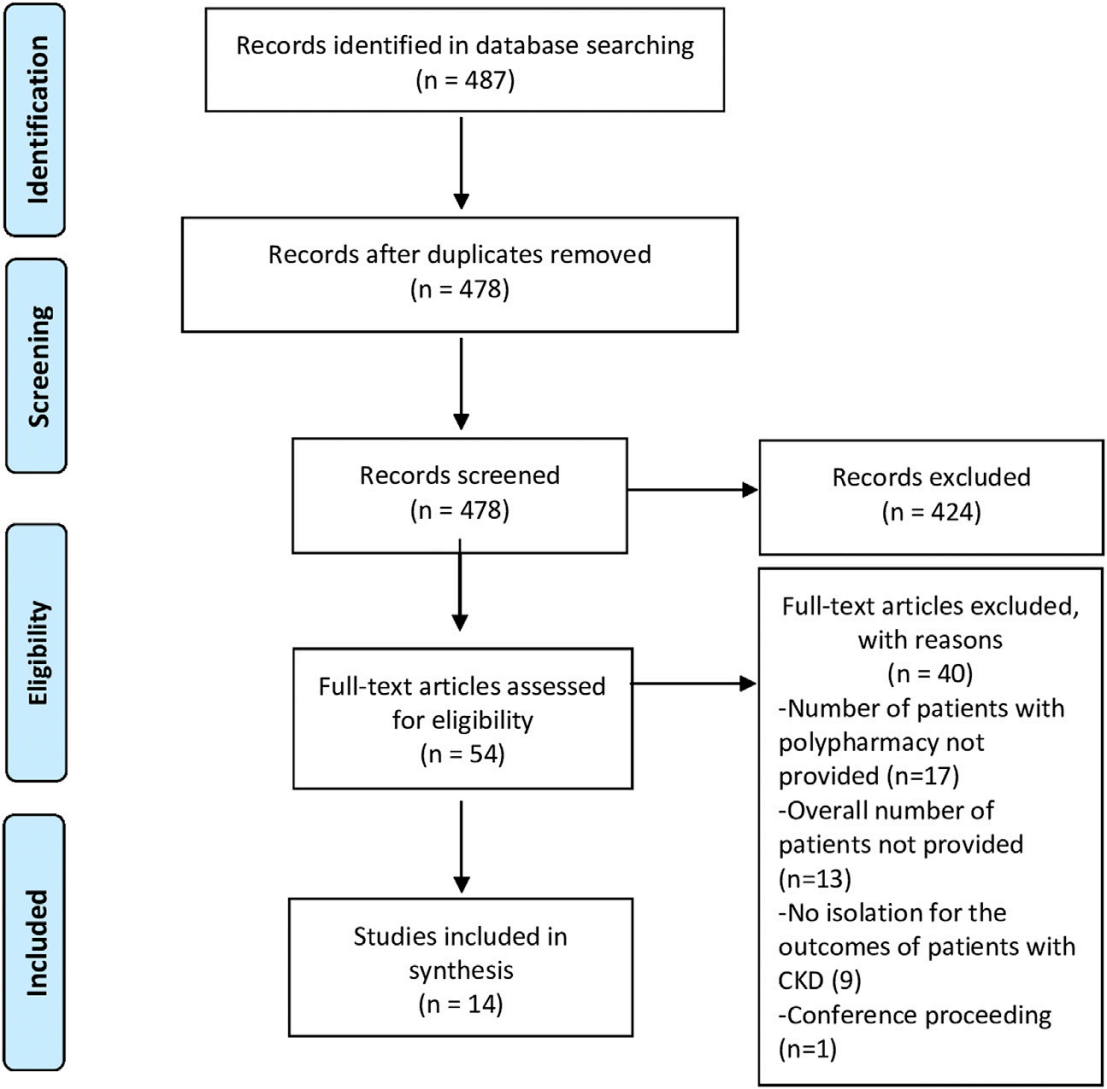
PRISMA flow diagram of the study selection process.

A total of 487 citations were retrieved from the literature search. After duplicate removal (*n* = 478), the remaining articles were screened by title and abstract. A total of 14 studies were included in the systematic review and meta-analysis (Figure 1). The most common reason for exclusion was the absence of data that allows the estimation of the prevalence of polypharmacy.

### Characteristics of included studies

Table 1 summarizes the characteristics of the included studies. The reviewed studies were distributed across four continents with three studies conducted in the USA (Bowling et al., 2014; Sutaria et al., 2016; Hawley et al., 2019), two in Germany (König et al., 2017; Schmidt et al., 2019), and one each in Canada (Battistella et al., 2018), Saudi Arabia (Meraya and Alwhaibi, 2020), Lebanon (Chahine, 2020), Italy (Cojutti et al., 2016), India (Subeesh et al., 2020), Korea (Min et al., 2021), Japan (Kimura et al., 2021), and Ethiopia (Garedow et al., 2019). The last study took place across multiple European countries (Hayward et al., 2020). Of the 14 studies included, six were undertaken in outpatient settings (clinics, community dwelling) (Bowling et al., 2014; König et al., 2017; Battistella et al., 2018; Hawley et al., 2019; Schmidt et al., 2019; Hayward et al., 2020), four in hospitals (Garedow et al., 2019; Chahine, 2020; Subeesh et al., 2020; Kimura et al., 2021), two in multiple settings (Cojutti et al., 2016; Min et al., 2021), and two did not report (Sutaria et al., 2016; Meraya and Alwhaibi, 2020). Most studies were retrospective or prospective cohort studies (*n* = 6) (Sutaria et al., 2016; Garedow et al., 2019; Hawley et al., 2019; Schmidt et al., 2019; Hayward et al., 2020; Min et al., 2021), followed by cross–sectional observational studies (*n* = 5) (König et al., 2017; Battistella et al., 2018; Chahine, 2020; Meraya and Alwhaibi, 2020; Subeesh et al., 2020), longitudinal studies (*n* = 2) (Bowling et al., 2014; Kimura et al., 2021), and point-prevalence study (*n* = 1) (Cojutti et al., 2016). The follow-up duration ranged from 6 months (Garedow et al., 2019; Chahine, 2020; Subeesh et al., 2020) to 10 years across included studies (Min et al., 2021).

**TABLE 1:** Characteristics of included studies.

Author, year of publication	Type of study	Age	Gender (male)	Site	Population characteristics	Country	Total number of patients with CKD	Case (CKD with polypharmacy)	Numerical description	Exact definition of polypharmacy in article	Duration
Battistella3 et al., (2018)	Retrospective cross-sectional study	Mean (±SD) 76.5 (±7.3)	55.6%	Outpatients	All patients were on hemodialysis. The 6 most common comorbidities were coronary artery disease (62%), diabetes (53%), heart failure (52%), chronic lung disease (42%), arrhythmia (29%), and atrial fibrillation (22%). The median duration of incenter hemodialysis was 3 (1–6) years	Canada	3094	2882	≥5	None	Not reported
Bowling et al., (2014)	Population-based longitudinal study	All patients were ≥75 years old	45.5%	Community dwelling	Black (33.6%). GFR≥60 (73%). GFR 45–59 (17%). GFR <45: 9.4%	United States	941	282	≥10	The concurrent use of ≥10 prescription medications	5 years
Chahine, (2020)	Retrospective cross-sectional study	Mean (±SD) 76.47 (±8.2)	46.2%	Two teaching hospitals	Patients were admitted for non-renal reasons (88.9%), median length of hospitalization: 8 days, hemodialysis (41.2%), hypertension (94%), coronary heart disease (72.4), diabetes (55.8%)	Lebanon	199	123	≥5	Using ≥5 medications per day	6 months
Cojutti et al., (2016)	Point-prevalence study	Median (IQR) Hospitals: 81 (75–87 Community: 76 (71–82) long-term care facilities (LTCF): 85 (79–89)	Hospitals: 50.6% community: 42.7% LTCF: 29.6%	Hospital (*n* = 528), community (*n* = 527), LTCF (*n* = 527)	≥5 underlying diseases: hospitals (24.8%), community (33.39%), LTCF (19.35%) Elderly (65–79 years): hospitals (39.6%), community (62.4%), LTCF: (22.9) Very elderly (>79 years): hospital (60.4%), community (37.6%), LTCF (77.1%)	Italy	1582	Polypharmacy patients: 1063 Hyperpolypharmacy: 213	Polypharmacy: ≥5 Hyperpolypharmacy: ≥10	Polypharmacy: the co-prescription of 5–9 drugs Hyperpolypharmacy: the co-prescription of ≥10 drugs	Not reported
Garedow et al., (2019)	Prospective observational study	Mean (±SD) 45.83 (±17.7)	69.9%	Hospital	Had <5 comorbidities (87.4%), newly diagnosed CKD patients (64.1%), stay in hospital for ≥7 days (77.7%), normal BMI (42.7%), CKD stage II (1.9%), CKD stage III (17.5%), CKD stage IV (15.5%), CKD stage V (65%)	Ethiopia	103	48	≥5	Use of ≥5 medication concomitantly	6 months
Hawley et al., (2019)	Pilot prospective cohort study	Mean (±SD): 73 (±10)	97%	Outpatient nephrology clinic	Non-dialysis kidney disease (89% stages III-V), hypertension (85%), dyslipidemia (80%), type II diabetes (59%)	United States	87	75	>10	Taking > 10 medications	10 months
Hayward et al., (2020)	Prospective cohort study	Mean (±SD) 76.5(±6.7)	64.2%	Nephrology clinics	The median eGFR was 18.0 mL/min/1.73m2 (IQR 16.0–19.0). All participants had at least one comorbidity in addition to CKD. The most frequent comorbidities were hypertension (84.4%), diabetes (42.4%), and coronary artery disease (27.3%). The mean BMI was 28.5 kg/m2 (±5.4)	Germany, Italy, Netherlands, Poland and the United Kingdom	1,317	Polypharmacy: 1194 Hyperpolypharmacy: 564	Polypharmacy: ≥5 Hyperpolypharmacy: ≥10	None	5 years
Kimura et al., (2021)	Retrospective longitudinal study	Median (IQR) 66 (58–75)	56%	Hospital	The median eGFR was 48 mL/minper1.73m2, participants with hypertension (87%), participants with diabetes (49%)	Japan	1,117	Polypharmacy: 429 Hyperpolypharmacy: 427	Polypharmacy: ≥5 Hyperpolypharmacy: ≥10	Polypharmacy: the regular use of 5–9 per day Hyperpolypharmacy: the regular use of ≥10 medications per day	Not reported
König et al., (2017)	Cross-sectional study	Mean (±SD) 68.7 (±3.7)	48.8%	Community dwelling	Hypertension (77.1%), diabetes (12.4%), obese (18.3%), GFR-CG, mL/min/1.73 m2 77.8 (49.5–122.2)	Germany	317	103	≥5	The use of ≥5 regular drugs, considering prescription and over-the-counter as well as scheduled and as-needed medications	6 years
Meraya and Alwhaibi, (2020)	Cross-sectional study	In all groups (not only CKD) Age (21–39): 6.5% Age (40–49): 12.9% Age (50–64): 37.9% Age (≥65):42.7%	In all groups (not only CKD) 49.3%	Not reported	In all groups (not only CKD): no chronic physical condition (11.8%), 1–2 chronic physical conditions (48.7%), 3–4 chronic physical conditions (30.8%), ≥5 chronic physical conditions (8.7%). Patients having mental conditions (13.8%). Obese (54.6%), overweight (29.8%), underweight/normal (14.1%)	Saudi Arabia	480	354	≥6	Use of ≥6 medication classes	6 years
Min et al., (2021)	Prospective cohort study	Mean (±SD) 53.6 (±12.3)	60.8%	Multicenter	Mean eGFR was 53.6 mL/min/1.73 m2. The causes of CKD were diabetic nephropathy (25.3%), hypertensive nephropathy (19.6%), glomerulonephritis (30.7%), and other causes (24.5%)	Korea	1,913	518	≥6	None	10 years
Schmidt et al., (2019)	Prospective observational study	Mean (±SD) CKD stage G1 41.8 (±12.9) CKD stage G2: 55.6 (±12.6) CKD stage G3a: 61.3 (±10.4) CKD G3b: 62.6 (±10.6) CKD stage G4/5: 63.5 (±10.1)	CKD stage G1 51.5% CKD stage G2: 54.4% CKD stage G3a: 62% CKD G3b: 61.5% CKD stage G4/5: 62.5%	Multiple outpatient units	CKD stage G3a (33.3%), CKD stage G3b (36.1%). Hypertension, diabetes, CVD and dyslipidemia were the most frequent comorbid conditions showing an increase in prevalence with lower eGFR	Germany	5,217	4,173	5 ≥	The daily use of ≥5 active substances, including the intake of non-oral and OTC medications	2 years
Subeesh et al., (2020)	Cross-sectional study	Mean (±SD) 50.08 (±15.32)	71.25%	Hospital	All patients had hypertension, diabetes (29.31%), anemia (11%), average comorbidities: 1.7 ± 1.86	India	160	146	>5	None	6 months
Sutaria et al., (2016)	Prospective study	≥60–69 (27.03%), ≥70–79 (55.76%), ≥80 (73.26)	43.11%	Not reported	Hypertension (50.74%), diabetes (54.13%), cardiovascular diseases (60.57%) 1,122.46	United states	674	308	≥5	The use of ≥5 prescription medications per day	1 year

Overall, a total of *N* = *17 201* participants were included in this systematic review and meta-analysis, of which 43.88% were females. Among studies that reported the average age (*n* = 9), the mean age was 61.96 (SD ± 11.51) years. All the constituent studies had patients with at least one medical condition alongside the CKD, with hypertension being the most frequently reported. Other common co-morbidities were diabetes, coronary artery disease, and other cardiovascular diseases (Table 1). The stage of the CKD amongst the reviewed studies was variable. For instance, Battistella et al. (2018) focused exclusively on patients on hemodialysis (stage 5, glomerular filtration rate (GFR) <15 mL/min), while the majority of patients in Bowling et al. (2014) study and Schmidt et al. (2019) were in stage 2 (GFR >60 mL/min) and stage 3 (GFR 30–60 mL/min), respectively.

### Quality of included studies

The overall quality of included studies was moderate to good mainly due to issues related to the sample size calculation and the appropriateness of outcome measures (Table 2).

**TABLE 2:** Quality assessment of included studies.

Author, year of publication	1. Was the design appropriate for the research question?	2a. Where setting of study and subjects described in detail?	2b. Are the subjects comparable to my population of interest?	3. Was subject sample obtained appropriately?	4. Was sample size appropriate?	5. Were objective and appropriate criteria used for measurement of outcomes?	6. Was outcome measured appropriately?	7. Are the estimates of prevalence precise?	8. Was response rate adequate?	Total
Battistella et al., (2018)	Yes	Yes	Yes	Yes	Yes	Cannot tell	Yes	Yes	Yes	7
Bowling et al., (2014)	Yes	Yes	Yes	Yes	Yes	Cannot tell	Yes	Yes	Yes	7
Chahine, (2020)	Yes	Yes	Yes	Yes	No	Cannot tell	Yes	Yes	Yes	6
Cojutti et al., (2016)	Yes	Yes	Yes	Yes	No	No	Yes	Yes	Yes	6
Garedow et al., (2019)	Yes	Yes	Yes	Yes	No	Cannot tell	Yes	Yes	Yes	6
Hawley et al., (2019)	Yes	Yes	Yes	Yes	No	Cannot tell	Yes	Yes	Yes	6
Hayward et al., (2020)	Yes	Yes	Yes	Yes	Yes	Cannot tell	Yes	Yes	Yes	7
Kimura et al., (2021)	Yes	Yes	Yes	Yes	Cannot tell	Cannot tell	Yes	Yes	Yes	6
König et al., (2017)	Yes	Yes	Yes	Yes	Cannot tell	Cannot tell	Yes	Yes	Yes	6
Meraya and Alwhaibi, (2020)	Yes	Yes	Yes	Yes	No	No	Yes	Yes	Yes	6
Min et al., (2021)	Yes	Yes	Yes	Yes	No	Cannot tell	Yes	Yes	Yes	6
Schmidt et al., (2019)	Cannot tell	Yes	Yes	Yes	Yes	Cannot tell	Yes	Yes	Yes	6
Subeesh et al., (2020)	Yes	Yes	Yes	Yes	Cannot tell	Cannot tell	Yes	Yes	Yes	6
Sutaria et al., (2016)	Yes	Yes	Yes	Yes	Yes	Cannot tell	Yes	Yes	Yes	7

### Prevalence of polypharmacy in patients with CKD

**FIGURE 2:**
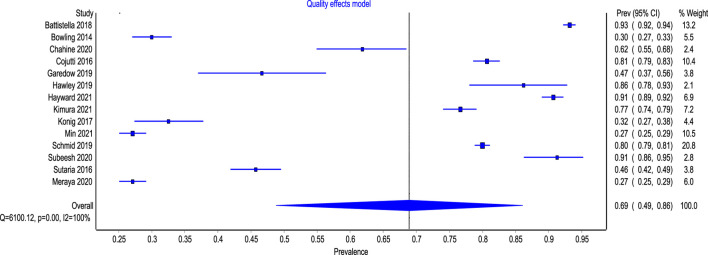
Forest plot of the prevalence of polypharmacy among patient with CKD.

Figure 2 presents the pooled estimates of the included studies. The overall pooled prevalence of polypharmacy amongst patients with CKD was 69% (95% CI: 49%–86%). The overall heterogeneity among the included studies was significant (I^2^ = 100%, *p* < 0.0001).

**FIGURE 3:**
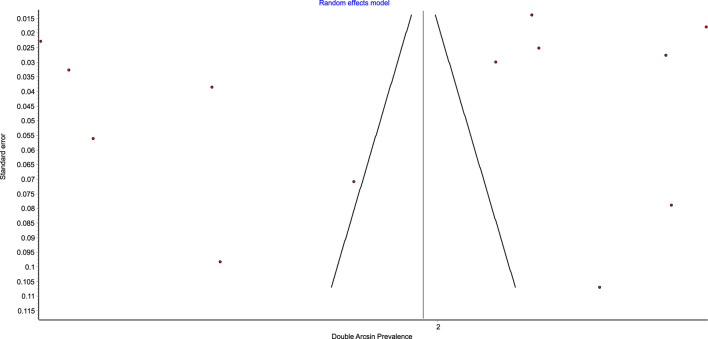
Funnel plot of the prevalence of polypharmacy among patient with CKD.

**FIGURE 4:**
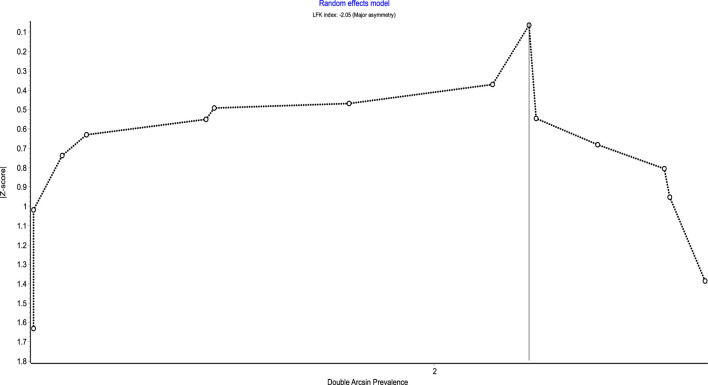
Doi plot of the prevalence of polypharmacy among patient with CKD.

Figures 3, 4 show the comparison adjusted funnel plot and Doi plot of the included studies. The plots demonstrated major (LFK 2.05) asymmetry which indicates major publication bias. This could also be attributed to the substantial between-study heterogeneity and small-study effect. No continuity correction was applied because the double arcsine prevalence transformation does not require one.

A sensitivity analysis was also performed by excluding one study each time and recalculating the pooled prevalence of polypharmacy for the remaining studies (Table 3). Based on this, the estimated pooled prevalence of polypharmacy in patients with CKD ranged between 61% and 67%.

**TABLE 3:** Point estimates of the various included studies following sensitivity analysis.

Excluded study	Pooled prevalence	LCI 95%	HCI 95%	Cochran Q	P	I^2^	I^2^ LCI 95%	I^2^ HCI 95%
Battistella et al. (2018)	0.61	0.45	0.77	4558.14	0.00	99.74	99.70	99.77
Bowling et al. (2014)	0.67	0.50	0.82	5453.33	0.00	99.78	99.75	99.80
Chahine (2020)	0.65	0.47	0.80	6095.05	0.00	99.80	99.78	99.82
Cojutti et al. (2016)	0.63	0.45	0.79	5979.91	0.00	99.80	99.77	99.82
Garedow et al. (2019)	0.66	0.49	0.81	6077.79	0.00	99.80	99.78	99.82
Hawley et al. (2019)	0.62	0.45	0.78	6085.90	0.00	99.80	99.78	99.82
Hayward et al. (2020)	0.62	0.44	0.78	5667.05	0.00	99.79	99.76	99.81
Kimura et al. (2021)	0.63	0.45	0.80	6067.64	0.00	99.80	99.78	99.82
Konig et al. (2017)	0.67	0.50	0.82	5917.24	0.00	99.80	99.77	99.82
Meraya and Alwhaibi (2020)	0.67	0.52	0.81	4479.19	0.00	99.73	99.70	99.76
Min et al. (2021)	0.67	0.52	0.81	4479.19	0.00	99.73	99.70	99.76
Schmid et al. (2019)	0.63	0.44	0.81	5657.46	0.00	99.79	99.76	99.81
Subeesh et al. (2020)	0.62	0.45	0.78	6048.37	0.00	99.80	99.78	99.82
Sutaria et al. (2016)	0.66	0.48	0.81	5937.59	0.00	99.80	99.77	99.82

### Global trend in the prevalence of polypharmacy in patients with CKD

**FIGURE 5:**
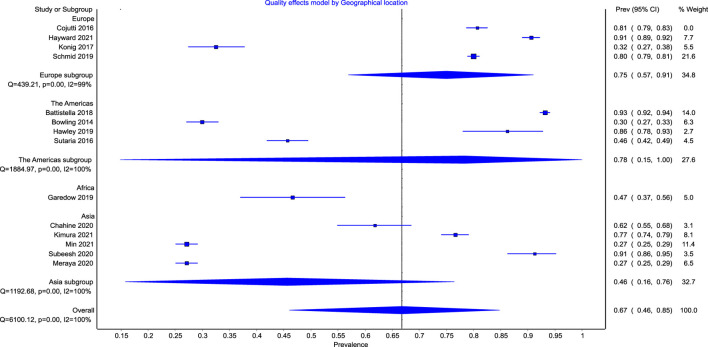
Forest plot of the prevalence of polypharmacy among patient with CKD across continents.

The subgroup meta-regression showed a higher pooled prevalence of polypharmacy of 78% (95% CI: 63%–92%) in Europe and 78% (95% CI: 15%–100%) in North America as compared with 48% (95% CI: 0%–100%) in Asia (Figure 5). The overall heterogeneity was significant for both overall analysis (I^2^ = 100%, *p* < 0.0001) and subgroup analyses (I^2^ = 99%, *p* < 0.01), (I^2^ = 100%, *p* < 0.01), (I^2^ = 100%, *p* < 0.01) for the European, North American, and Asian studies, respectively.

Only one study was identified from Africa (Garedow et al., 2019), therefore estimating the pooled prevalence was not feasible. Similarly, we could not calculate the prevalence in South America, Antarctica, or Australia as no relevant studies were identified from these continents.

## Discussion

To the best of our knowledge, this systematic review and meta-analysis represents the first comprehensive synthesis of the prevalence of polypharmacy and the global trends associated with its variability among patients with CKD. We explored the longitudinal data of 17,201 patients from 13 countries in 4 continents. The overall pooled prevalence of polypharmacy amongst patients with CKD was 69% with a proportionately higher prevalence in North America and Europe as compared to Asia. Despite the apparent disparity in the stage of CKD amongst the included studies, our period prevalence estimate provides the first attempt at exploring the burden of this growing therapeutic morbidity in this cohort of patients.

The prevalence estimates reported in this review are substantially higher than the general population including cohorts at high risk of experiencing polypharmacy such as elderly (Veehof et al., 2000), patients with chronic liver disease (Danjuma et al., 2022a), and people living with HIV (Danjuma et al., 2022b). However, the estimates were comparable to other populations that are also known to be more exposed to polypharmacy such as heart failure patients (Beezer et al., 2021).

This remarkably high prevalence is alarming particularly in CKD patients as they represent a more challenging and therapeutically vulnerable population; principally due to the central role of the kidney in drug metabolism (including other aspects of pharmacokinetics and pharmacodynamics) (Laville et al., 2020). This therefore imposes higher risk of adverse drug reactions (ADR) and their sequalae on patients with CKD (Laville et al., 2020).

Additionally, patients included in our review had an average age of 61.96 (SD ± 11.51) years, which is expected as CKD in more prevalent in people older than 60 years old (Aging and Kidney, 2022). This further complicates the situation as there is mounting evidence of the increased risk of mortality in response to increased drug counts, especially in the elderly (Patel et al., 2017; Davies et al., 2022). For instance, the Newcastle 85 + study showed that for each additional medication prescribed in patients aged 85 years and older, there is a 3% associated risk of increased mortality (hazard ratio: 1.03, 95% CI: 1.00–1.06) (Davies et al., 2022).

Our findings underscore the gravity of the rising burden of polypharmacy among patients with CKD and highlight the pressing need to adopt some of the interventions that have been proposed to reduce the burden of polypharmacy in the general population. This includes comprehensive medication reviews, deprescribing algorithms, potentially inappropriate medications (PIM) screening tools (e.g., Beers criteria), and clinical pharmacist-led interventions (Cooper et al., 2015; El‐Awaisi et al., 2022; Isleem et al., 2022). It is noteworthy that these interventions are not widespread and that the research on the effectiveness of such strategies on clinically important outcomes is limited (Cooper et al., 2015). Hence, future research efforts should focus on measuring the effectiveness of these interventions. Moreover, experts should attempt at developing multifaceted theory-based interventions that are tailored to patients with CKD. This approach is expected to yield promising outcomes as other methods of interventions development [i.e., pragmatic approach or ISLAGIATT (It Seemed Like A Good Idea At The Time) principle] were checkered, with some showing unfavorable outcomes or no benefit at all (Michie et al., 2005; Hughes et al., 2016; Steinmo et al., 2016).

### Strengths and limitations

The principal strength of this synthesis lies in its novelty at estimating the overall pooled prevalence of polypharmacy among patients with CKD. This will enable clinicians, as well as policy makers, and other stakeholders to more robustly estimate the burden of polypharmacy and more appropriately allocate intervention strategies aimed at mitigating their downstream effects (including bidirectional interactions as well as adverse drug reactions). The review also involved comprehensive searching of three large databases using established methods and was reported following a standardized method. Despite this, there were several limitations. Firstly, only English language publications were included. Secondly, polypharmacy prevalence was not the primary outcome measure in many of the included studies which resulted in the lack of in-depth information relating to it. Finally, significant heterogeneity was noted across the included studies. This could be attributed to the variation in the definition of polypharmacy among included studies. Other factors include the various study design, the geographical disposition of areas where the studies were carried out, and the difference in the mean age of the constituent studies.

## Conclusion

The results from this meta-analysis showed a high pooled prevalence estimates of polypharmacy amongst patient cohorts with CKD. The exact interventions that are likely to significantly mitigate its effect remain uncertain and will need exploration by future prospective and systematic studies.

## Data Availability

The original contributions presented in the study are included in the article/supplementary materials, further inquiries can be directed to the corresponding author.
